# An Integrated Approach to Testing and Assessment to Support Grouping and Read-Across of Nanomaterials After Inhalation Exposure

**DOI:** 10.1089/aivt.2021.0009

**Published:** 2021-09-17

**Authors:** Hedwig M. Braakhuis, Fiona Murphy, Lan Ma-Hock, Susan Dekkers, Johannes Keller, Agnes G. Oomen, Vicki Stone

**Affiliations:** ^1^ Centre for Health Protection and Centre for Safety of Substances and Products, National Institute for Public Health and the Environment (RIVM), Bilthoven, The Netherlands.; ^2^ NanoSafety Research Group, Heriot Watt University, Edinburgh, United Kingdom.; ^3^ Experimental Toxicology and Ecology, BASF, Ludwigshafen am Rhein, Germany.

**Keywords:** case study, grouping, IATA, inhalation exposure, nanomaterials, read-across, testing strategy

## Abstract

***Introduction:*** Here, we describe the generation of hypotheses for grouping nanoforms (NFs) after inhalation exposure and the tailored Integrated Approaches to Testing and Assessment (IATA) with which each specific hypothesis can be tested. This is part of a state-of-the-art framework to support the hypothesis-driven grouping and read-across of NFs, as developed by the EU-funded Horizon 2020 project GRACIOUS.

***Development of Grouping Hypotheses and IATA:*** Respirable NFs, depending on their physicochemical properties, may dissolve either in lung lining fluid or in acidic lysosomal fluid after uptake by cells. Alternatively, NFs may also persist in particulate form. Dissolution in the lung is, therefore, a decisive factor for the toxicokinetics of NFs. This has led to the development of four hypotheses, broadly grouping NFs as instantaneous, quickly, gradually, and very slowly dissolving NFs. For instantaneously dissolving NFs, hazard information can be derived by read-across from the ions. For quickly dissolving particles, as accumulation of particles is not expected, ion toxicity will drive the toxic profile. However, the particle aspect influences the location of the ion release. For gradually dissolving and very slowly dissolving NFs, particle-driven toxicity is of concern. These NFs may be grouped by their reactivity and inflammation potency. The hypotheses are substantiated by a tailored IATA, which describes the minimum information and laboratory assessments of NFs under investigation required to justify grouping.

***Conclusion:*** The GRACIOUS hypotheses and tailored IATA for respiratory toxicity of inhaled NFs can be used to support decision making regarding Safe(r)-by-Design product development or adoption of precautionary measures to mitigate potential risks. It can also be used to support read-across of adverse effects such as pulmonary inflammation and subsequent downstream effects such as lung fibrosis and lung tumor formation after long-term exposure.

## Introduction

Manufacturing and functionalizing of materials at the nanoscale leads to an array of nanoforms (NFs) of each nanomaterial (NM), which may vary in physicochemical (PC) properties such as chemical composition, size, morphology, and surface characteristics. The definitions of an NM and an NF as given by the European Commission are shown in the Supplementary Data (Supplementary Table S1). Apart from expected benefits, modification of NFs may also pose a hazard to human health to a greater or lesser extent than the unmodified NF. Risk assessment requires comprehensive PC characterization as well as sufficient exposure and hazard data for each NF, but testing every unique NF for their potential adverse effects would demand substantial resources, including large numbers of animals.

Grouping and read-across are evolving into important tools in the safety assessment of chemical substances, including NFs. Formation of a group requires the properties of the grouped substances to be similar or to follow a consistent trend. For chemical substances, grouping is typically based on evidence of similar chemical structures, common functional groups, common precursors, or likely common breakdown products (REACH, Annex XI, 1.5 and Organization for Economic Co-operation and Development [OECD] guidance).^1^ Read-across allows prediction of specific fate and hazard endpoints for one or more substances (target material(s)) in a group, by using data for the same endpoint from another substance in the same group for which more information is available (source material).^2^ This approach can be used to fill data gaps where hazard data are lacking, thereby minimizing the need to perform additional *in vivo* studies for each group member. Grouping of NMs typically involves the grouping of different NFs of one chemical substance or the grouping of a nano- and a non-NFs(s) of one chemical substance. It requires similarity in PC parameters with known relevance for human and environmental hazards. Key intrinsic material characteristics as highlighted in the ECHA guidance for grouping NMs (Appendix R.6–1) include chemical composition, impurities, and functionalization, in addition to particle size, shape, and surface area.^3^ System-dependent properties governed by the surroundings in which the NF is placed (e.g., dissolution rate in biological media, surface reactivity, and dispersibility) should also be considered to support grouping.^4^

In recent years, several scientific approaches for grouping and read-across of NFs have been developed.^5–9^ The EU-funded Horizon 2020 project, GRACIOUS has taken these approaches a step further by developing a state-of-the-art framework to support the hypothesis-driven grouping of NFs and streamline the risk assessment process.^10^ Read-across between NFs of the same group can be utilized as an efficient and effective tool to obtain toxicological information and fill data gaps without resorting to animal testing of individual NFs for hazard assessment, including for a regulatory setting. Within the GRACIOUS framework a number of “pre-defined” grouping hypotheses have been generated, based on clear toxicokinetic pathways or mechanisms of action. These allow the user to quickly recognize a potential hazard, which may be applicable to the NF(s) under investigation.^10^

GRACIOUS has also developed tailored Integrated Approaches to Testing and Assessment (IATA), which gather evidence to justify (or reject) grouping of a target NF and a source material. The IATA sets out a tiered testing strategy, which reflects the different information needed and levels of uncertainty acceptable for different grouping purposes. Here, we propose a number of purposes for which the use of the inhalation IATA will be appropriate:

(1) Grouping to guide and support the development of materials and NFs that are Safe(r) by Design (SbD).
(2) Grouping to promote the adoption of precautionary measures for materials for which limited hazard data are available.
(3) Facilitating the generation of a read-across argument for filling in a data gap to comply with regulations.

The substantiation of a grouping decision is underpinned by the demonstration of similarity between group members, which helps the user to assess whether a target NF is sufficiently similar to a source material to allow grouping and to assume the target NF will induce similar toxicity compared with the source material. For SbD, for the adoption of precautionary measures, and for screening whether regulatory read-across could be possible, a qualitative similarity assessment based on expert judgment is sufficient. For regulatory read-across, quantitative mathematical similarity assessment is necessary to compare the NF with the source material. Here, we describe the generation of GRACIOUS “pre-defined” hypotheses for grouping NFs where inhalation exposure is a primary concern, and the tailored IATA to test each specific hypothesis. The use of the IATA, including qualitative similarity assessment, will be demonstrated by using benchmark materials.

### Grouping hypotheses

Within the GRACIOUS framework, the user is first asked for basic information to identify the NFs under consideration and their potential uses to identify the most appropriate hypotheses to test.^10^ In addition, the basic information gathers information needed to tailor the outputs of the grouping and read-across exercise to the purpose of grouping. According to the GRACIOUS framework, the basic information therefore requires the user to identify: the purpose of grouping, basic PC characteristics, and the use/exposure scenarios.

Four hypotheses have been generated for grouping NFs with predicted similar fate and a subsequent assessment of similar hazard following the inhalation route of exposure (Table 1). The hypotheses include both acute and repeated exposure.

**Box 1.** Potential IATA Outcomes

• Accept grouping hypothesis and use outcome for SbD of new NFs.
• Accept grouping hypothesis and use to design precautionary measures by assuming target NF will cause similar long-term effects compared with the source NF.
• Accept grouping hypothesis and then progress to building a read-across argument (the final similarity may still be unacceptable).
• Reject grouping hypothesis, because the NFs dissolve at different rates, which may lead to different toxicokinetics (and therefore different bioaccumulation and long-term effects)
• Reject the grouping hypothesis, because one appears much more reactive (more potent) than the other or produces ROS/oxidative stress due to a different mechanism of action.
• Reject the grouping hypothesis, because one appears much more inflammogenic than the other (more potent).

**Table 1.** GRACIOUS “Pre-Defined” Hypotheses for Inhalation Exposure to Nanoforms

Short title	Hypothesis
**I**nstantaneously dissolving NFs (H-I-**I**)	Respirable NFs with an instantaneous dissolution rate: After inhalation exposure, the toxicity is driven by and is therefore similar to those of the constituent ions or molecules.
**Q**uickly dissolving NFs (H-I-**Q**)	Respirable NFs with a quick dissolution rate: After inhalation exposure, both NFs and constituent ions or molecules may contribute to toxicity, but there is no concern for accumulation. Toxicity (also) depends on the location of the ionic or molecular release.
**G**radually dissolving NFs (H-I-**G**)	Respirable NFs with a gradual dissolution rate: After inhalation exposure, both NFs and constituent ions or molecules may contribute to toxicity and there is some concern for accumulation. Toxicity (also) depends on the location of the ionic or molecular release.
Very **s**lowly dissolving NFs (H-I-**S**)	Respirable NFs with a very slow dissolution rate: After inhalation exposure, toxicity is driven by the NFs and accumulation of NFs in the lungs can lead to long-term toxicity.

H-I-G, hypothesis for inhaled NFs that gradually dissolve; H-I-I, hypothesis for inhaled NFs that instantaneously dissolve; H-I-Q, hypothesis for inhaled NFs that quickly dissolve; H-I-S, hypothesis for inhaled NFs that very slowly dissolve; NFs, nanoforms.

### Dissolution as a critical descriptor

Information on the use and most relevant exposure route of the NF is gathered as part of the basic information at the start of the GRACIOUS framework and guides the user as to whether inhalation exposure is expected.^10^ Each of the four inhalation hypotheses is shortlisted within the GRACIOUS framework when the aerosolized NFs under investigation are within the respirable range (<4.2 μm).^11^ On deposition in the respiratory tract, NFs first come into contact with mucus in the upper respiratory tract and lung lining fluid (LLF) (pH7.4) in the deeper lung, respectively. Depending on the PC properties of the specific NFs, they may dissolve either in mucous and LLF, or in acidic phagolysosomal fluid (PLF) (pH 4.5) after uptake by cells, or persist within the lung, interstitium, or lung-associated lymph nodes for an extended period of time. Deposited particles within the upper respiratory tract and tracheobronchial tree are cleared by different mechanism, including mucociliary transport, within the first hours.^12^ Our grouping hypotheses are concerned with the fate and potential hazard posed by NFs that reach the distal regions of the lung, where accumulation may occur, leading to chronic adverse effects in the local tissue as this context is considered the primary concern after inhalation exposure of NFs.

There are several approaches for grouping and read-across, which identify dissolution under simulated physiological conditions as a crucial criterion for grouping and subsequent read-across between NFs.^5,8,13–17^ Oberdörster and Kuhlbusch describe in their recent review that “because the *in vivo* dissolution rates of engineered nanomaterials can differ widely, it is too simplistic to group ENM just into soluble and poorly soluble materials.”^18^ There are currently no scientifically sound cut-off thresholds to define groups according to dissolution rate, as the transition from very slow to quick dissolution rate is continuous. However, here the following pragmatic thresholds are suggested to facilitate the preliminary grouping of NFs into broad categories:

(1) Instantaneously dissolving NFs: threshold of t_1/2_ <10 minutes in LLF (H-I-I).
(2) Quickly dissolving NFs: threshold of t_1/2_ <48 hours in lung lining or lysosomal fluid (H-I-Q).
(3) Gradually dissolving NFs: threshold of t_1/2_ >48 hours and <60 days in lung lining or lysosomal fluid (H-I-G).
(4) Very slowly dissolving NFs: threshold of t_1/2_ >60 days in lysosomal fluid (H-I-S).

In this article, we refer to instantaneously, quickly, gradually, and very slowly dissolving NFs to describe the dissolution rate by which NFs release ions/molecules/atoms and thereby alter their (physical) state or entity.

The pragmatic thresholds are set to reflect the impact of dissolution within the biologically relevant timeframe for cell interaction and cellular clearance from the lungs; for example, “instantaneous” dissolution within 10 minutes suggests that NFs do not persist long enough to be phagocytosed by alveolar macrophages or translocate through the epithelial barrier, therefore particle-triggered hazard is negligible. Alternatively, a longer half-life in LLF indicates the potential for particle–cell interactions and uptake of NFs into the lysosomal compartment of the resident pulmonary cells, which may ultimately trigger particle-related toxicity. The grouping hypotheses, H-I-Q, H-I-G, and H-I-S, address a number of biological outcomes that may result from differing half-lives within the LLF and/or acidic environment of the lysosome.

Quick dissolution (defined as a half-life of <48 hours in lysosomal fluid) reflects a timeframe whereby NFs may be taken up by cells, in particular alveolar macrophages, but they dissolve rapidly to constituent ions within the acidic environment of the lysosome.^19^ This mechanism directly delivers potentially toxic ions to the intracellular environment, which may lead to specific toxic effects such as cell death or activation of pro-inflammatory pathways.^20,21^ Accumulation of particles is not likely due to their quick dissolution and so direct toxicity driven by ions will be most relevant.

Gradual dissolution considers both the particles and NFs, which may persist in particulate form for some time but gradually degrade in either the LLF or the acidic lysosomal environment to their constitute components, indicating a slow release of ions over time.^22^ If exposure exceeds the dissolution and clearance rates of the particle components, NFs may potentially accumulate within the lungs.^22,23^ Thus, toxicity may be driven by both ion and particle effects and may incorporate both direct effects due to toxic ion release or highly reactive particle surface, as well as chronic effects due to the slow release of ions over time. Therefore, for quickly and gradually dissolving NFs, the IATA considers both the dissolved and the particulate fraction of the NFs under investigation.

Very slow dissolution is defined by a threshold half-life >60 days in lysosomal fluid, derived from the extensive literature on the biopersistence of poorly soluble particles in the rat lung.^24–26^ Biopersistent NFs will remain as particles in the pulmonary environment over an extended period of time and may accumulate in cells and tissue. Toxicity will be dictated by physical interactions between the NFs and cells, such as through excessive build-up of NFs^24,27^ or specific NF reactivity.^28^

According to Geiser and Kreyling (2010), about 90% of very small particles deposited in the alveolar region are cleared by alveolar macrophages, which are subsequently eliminated via mucociliary clearance. Other particle clearance pathways from the lung are via the interstitium and lymphatic system, through re-appearance of particles from the interstitium onto the epithelial surface and via translocation to the blood (potentially leading to accumulation in secondary target organs).^26,29,30^ On repeated exposure to biopersistent NFs, the clearance mechanisms can be overwhelmed, leading to NF accumulation and chronic inflammation, which might ultimately lead to fibrosis and/or cancer.^24^ Therefore, the targeted testing for these NFs differs significantly from those particles that instantaneously dissolve, by focusing on particle-triggered toxicity, including biopersistence, potential accumulation, and long-term effects.

### Biological reactivity as a critical descriptor

The mechanism of particle-induced toxicity is not yet fully understood. The previously described concept of impaired clearance does not explain the different inflammatory potencies of different NFs. Current research shows that a range of intrinsic factors such as shape, size, coating, composition, crystallinity, impurities,^15,31–33^ and extrinsic factors such as pH, temperature, ionic strength, and protein binding may modulate the surface reactivity of NFs. Thus, surface reactivity was considered as an essential parameter for building and justifying a grouping strategy for very slow, gradual, and quick dissolution NFs.

Several approaches to grouping and read-across acknowledge surface reactivity, such as reactive oxygen species (ROS) production, as a key parameter.^5,8,9^ The imbalance between ROS generation and ROS scavenging leads to elevated ROS levels within cells, non-selective oxidation of biomolecules,^34,35^ and oxidative stress associated with endpoints such as cytotoxicity, genotoxicity, or inflammation.^36–40^ The induction of oxidative stress (via ROS induction and inflammation) is believed to play an essential role in the mechanism behind NM toxicity.^41–48^

For grouping and read-across, it is insufficient to assign NFs into either a “not reactive” or “reactive” category as the level of ROS production by NFs can differ greatly. It is, therefore, essential to take the potency of NFs into account to substantiate a read-across argument.

### Inflammatory potential as critical descriptor

A key effect of NFs after inhalation is their ability to induce pulmonary inflammation.^24,49–52^ Inflammation is considered an important mechanism of action by which NFs may cause toxicity.^53^ It is related to various adverse outcomes that have been associated with NF exposure, including pulmonary fibrosis and cancer.^25,54^ Inflammation is indicated *in vivo* mainly by an increase in neutrophils and pro-inflammatory cytokines in the bronchoalveolar lavage fluid or via histopathological examination. In *in vitro* lung models, inflammation is generally indicated by the induction of pro-inflammatory cytokines.^15,55^

Inflammation is a complex process involving many cell types, chemokines, and cytokines. Also, depending on the exposure concentration and duration, inflammation can resolve over time. For NF exposure, the main concern is that repeated exposure might lead to chronic inflammation that does not resolve. Given the complexity of inflammation, it is not sufficient to categorize NFs into “inflammogenic” or “non-inflammogenic.” Similar to reactivity, the potency of the target and source NFs in terms of inflammation potential should be compared to assess their similarity to allow grouping and the subsequent building of a read-across argument.

## Integrated Approaches to Testing and Assessment

**FIG. 1.**
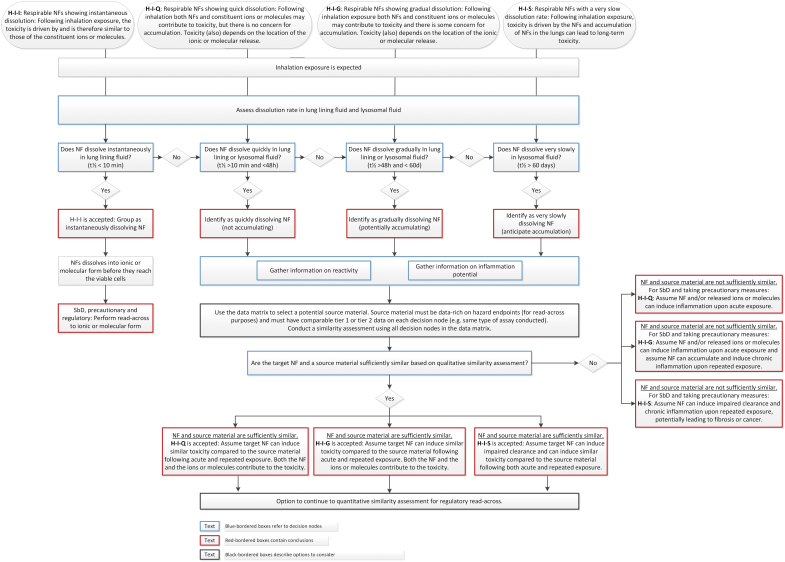
IATA decision tree for assessing whether an NF belongs to the group of instantaneously dissolving, quickly dissolving, gradually dissolving, or very slowly dissolving NFs, including directions on the implications of grouping and subsequent options for read-across. IATA, Integrated Approaches to Testing and Assessment; NF, nanoform. Color images are available online.

The GRACIOUS IATA is structured in a decision tree format, which logically follows the fate of the NFs from the initial inhalation exposure to deposition along the respiratory tract and the subsequent potential for interactions with resident pulmonary cells, which may lead to toxicity and disease pathogenesis. The decision tree uses a series of decision nodes (DNs) to generate the information needed for critical descriptors to selectively distinguish NFs, which may be grouped according to the specific inhalation grouping hypotheses (Fig. 1).

The hypothesis for instantaneously dissolving NFs (H-I-I) can be used to perform read-across to the molecular form. If the hypothesis is rejected because the NF does not meet the threshold of t_1/2_ < 10 minutes in LLF, then other hypotheses can be considered in which the location of the ion release will affect the toxicity (Fig. 1).

For NFs that quickly dissolve (t_1/2_ < 48 hours), there is no concern for particle accumulation and toxicity can mainly be attributed to the ions. However, the particle aspect influences the location of the ion release. A benchmark material that fits into this hypothesis is ZnO (JRCNM01100a, formerly known as NM-110). For ZnO NFs, the particles can be taken up by cells, leading to intracellular ion release^56^ referred to as the Trojan horse effect.^20,21^ This leads to different effects compared with exposure to zinc salts.^57–60^

For gradually dissolving NFs (t_1/2_ > 48 hours and <60 days in lysosomal fluid), both the particle and the ions contribute to the toxicity, and the location of ion release affects toxicity. As the dissolution rate is not quick, particle accumulation cannot be discounted for upon repeated exposure. A benchmark material that falls into this hypothesis is synthetic amorphous silica, SiO_2_ (JRCNM02000a, formerly known as NM-200).^23^ This material has a half-time of 3.6–4.5 days in LLF and 29–35 days in PLF and has been shown to induce inflammation after intratracheal instillation (IT).^61^ As toxicokinetics are important in this hypothesis, comparison to a source material of similar chemical composition to the NF(s) under investigation is needed for read-across.

Very slowly dissolving NFs (t_1/2_ > 60 days) are of concern, as they can accumulate and may induce long-term effects on repeated exposure. Benchmark materials that fit into this hypothesis are cerium dioxide (CeO_2_) (JRCNM02102a, formerly known as NM-212), DQ12 quartz silica, and TiO_2_ (JRCNM01005a, formerly known as NM-105). The dissolution rate of these materials is very slow and they are known to induce long-term effects in rats on repeated exposure. CeO_2_ JRCNM02102a induced chronic inflammation and fibrosis after 90 days of inhalation exposure.^62^ DQ12 quartz induced chronic inflammation and fibrosis after 90 days of inhalation exposure^63^ and cancer after chronic 2-year inhalation exposure.^64^ TiO_2_ JRCNM01005a induced chronic inflammation and cancer after chronic 2-year exposure.^25,27^ These long-term effects are related to impaired clearance in rats at high exposure concentrations caused by extensive accumulation of the particles. Intensive discussions are ongoing about the human relevance of these effects. From a risk assessment point of view, the pulmonary toxicity needs to be considered relevant for human hazard assessment.^24^

### Tiered testing

**FIG. 2.**
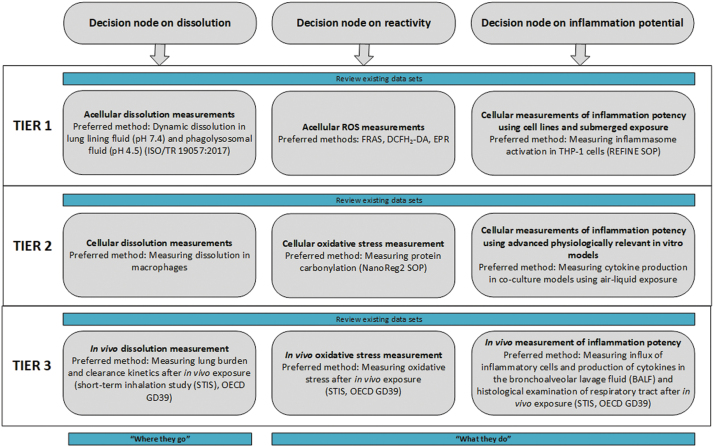
Tiered testing for each DN in the IATA for hypotheses H-I-Q, H-I-G, and H-I-S. DN, decision node; H-I-G, hypothesis for inhaled NFs that gradually dissolve; H-I-I, hypothesis for inhaled NFs that instantaneously dissolve; H-I-Q, hypothesis for inhaled NFs that quickly dissolve; H-I-S, hypothesis for inhaled NFs that very slowly dissolve. Color images are available online.

Each DN of the IATA is linked to a tiered testing strategy, which provides practical guidance on how to efficiently assess the target NF (Fig. 2). The testing strategy is tiered to enable the burden of data gathering and testing to be tailored to the purpose of grouping, with higher tiers reflecting the greater information requirements to support a grouping decision with higher levels of confidence.^10^ The choice of tier reflects the initial purpose for grouping, the associated level of uncertainty considered acceptable for the user's needs, and sometimes the suitability of the recommended methods for the NF under investigation. Lower tier testing may facilitate rapid and cost-effective SbD decision making on whether to continue with a product development, despite the relatively high level of uncertainty with this grouping decision. On the other hand, grouping and read-across for regulatory purposes may require a higher degree of scientific justification based on higher tier testing. When available, standardized methods (standard operating procedures [SOP] such as OECD TG or ISO protocols) are recommended for inclusion in the tiered testing strategy.

The tiered testing strategy provided in Figure 2 addresses the particle-induced hazard in the lung and is relevant to the IATA for hypotheses H-I-Q, H-I-G, and H-I-S. It is less relevant to H-I-I, since this hypothesis is addressed by assessing the hazard of the constituent ions or molecules. The outcome of the tiered testing strategies provides the required information needed by the DNs of the IATA to identify which hypothesis is most appropriate for grouping the NFs under investigation. The following sections provide a more detailed description of each DN.

### Dissolution DNs

The first DN in the IATAs is on the dissolution rate of the NF. Tier 1 testing for this DN includes assessment of the NFs dissolution in simulated LLF at pH 7.4 and PLF at pH 4.5 under static or dynamic conditions. Tier 2 testing for the dissolution DN includes measurement of durability in cellular systems such as macrophages. Tier 3 consists of *in vivo* measurement of lung burden and clearance kinetics.

Considering that living organisms are dynamic systems, static solubility tests do not reflect the *in vivo* turnover of the respective physiological media. Testing NF dissolution in an acellular continuous flow system is considered the preferred method in Tier 1, as the results of the continuous flow system are consistent with data from short-term *in vivo* studies.^65^ Standardized ISO protocols for these flow-through or flow-by systems that mimic the non-equilibrium physiological conditions are available (ISO/TR 19057:2017). Simulant media need to be sufficiently complex to offer oxidative, reductive, and pH-driven dissolution pathways.^66^ For inhalation exposure, both LLF and PLF are relevant media.^67^

Tier 2 examines the durability in cellular systems, which take into account a number of dynamic and physiologically relevant environments and pathways to NF degradation.^68–70^ As cellular models to assess durability are not yet well standardized, there is currently no SOP available, however, studies have shown incubation with macrophages to be at least as predictive of biodurability as acellular assays for NFs^65^ and useful to clarify the specific mechanism of particle degradation.^71^ As such, progression to Tier 2 is envisioned to be only used in some cases where a more physiologically relevant cellular system is required to better understand mechanisms.

The determination of biopersistence of NFs requires long-term *in vivo* assays and therefore is not required for initial grouping. Depending on the purpose of grouping, Tier 3 testing may be required to confirm whether acellular *in vitro* durability corresponds with an accumulation of NFs in tissues. For this, a short-term inhalation study (STIS) can be used with a 5-day exposure period and a recovery time of, for example, 28 days for very slowly dissolving NFs. The updated OECD test guidelines for inhalation exposure now recommend lung burdens and clearance rate to be included as recorded endpoints.^72,73^ To support grouping of NFs at Tier 3 the IATA requires clearance rate to be included as an endpoint, to provide evidence of similarity in biopersistence. This information can be used in case available for the source material.

Application of the tiered testing strategy to assess dissolution allows the NFs to be placed into one of four groups: instantaneously dissolving, quickly dissolving, gradually dissolving, and very slowly dissolving.

### Reactivity DNs

For the reactivity DN, Tier 1 assessment relies on acellular measures of ROS generation, Tier 2 includes measurement of ROS/oxidative stress in cells, and Tier 3 includes *in vivo* measurement of oxidative stress.

A panel of several acellular tests considered appropriate as a starting point to assess reactivity are included at Tier 1. They include ferric reduction ability of serum (FRAS), electron paramagnetic resonance (EPR), and dichlorodihydrofluorescin diacetate assay (DCFH_2_-DA). The FRAS assay uses antioxidant components in human serum as reporter molecules, providing an indirect read-out of ROS generation. The assay has been demonstrated to be suitable for testing both metal-containing NFs and carbonaceous materials.^74^ The EPR spectroscopy, which is also called electron spin resonance, measures the transition between electron spin states of paramagnetic molecules, and it can be used to study species with at least one unpaired electron. Using different spin probes, spin traps, different types of ROS species can be quantified. The EPR has the least interference with hydrophobic and colored substances; however, carbonaceous materials can interfere with the assay. DCFH_2_-DA assay can be used in Tier 1 to assess acellular ROS production. This assay has been widely used to assess the ROS production of particles and NFs.^75^ DCFH_2_-DA assay is suitable for the testing of carbonaceous materials.^74^

Different approaches to Tier 1 assessment of surface reactivity may be taken dependent on the purpose of grouping. For example, for SbD purposes where the aim may be to compare similarity of surface reactivity across NFs of different chemical composition or NFs with the same core and a different coating, a combination of assays would be recommended for a broader assessment of reactivity. Conversely, for grouping NFs for regulatory purposes, such as the development of a read-across argument, a comparison of surface reactivity of different NFs or non-NFs via a combination of assays might add unnecessary complexity. Therefore, a single assay that is sensitive to the substance-specific reactivity should be selected.^74^

Tier 2 involves cellular assessment of oxidative stress as a biological consequence of NF reactivity. More work is required to confirm the most appropriate tests to be incorporated into this tier. Currently, assays such as cellular DCFH_2_-DA assay, protein carbonylation, Nrf2 antioxidant response pathway, endoplasmic reticulum stress, heat shock protein activation, glutathione depletion, and lipid peroxidation are recommended for inclusion. Measuring protein carbonylation in cells has been shown to give a similar ranking of NFs compared with adverse reactions (such as inflammation) after *in vivo* short-term inhalation studies (STIS).^76^ Measuring glutathione depletion showed a correlation between *in vitro* and *in vivo* exposure for amorphous silica nanoparticles.^77^ The disadvantage of measuring glutathione is that it is easily reduced during sample preparation, making it difficult to assess the reduced and the oxidized form. An alternative method could be the use of antioxidants to assess whether specific endpoints (e.g., cytokine production) are oxidant mediated.

If Tier 3 *in vivo* studies are required to enable a grouping decision or to facilitate a read-across argument, measuring glutathione depletion and lipid peroxidation after short-term inhalation can be considered. In addition, endpoints such as oxidative DNA damage (by measuring 8-hydroxy-2-deoxyguanosine [8-OH-dG]) may be included in the histopathological assessment of tissue to provide evidence of oxidative stress *in vivo.*^38^

For NFs that are considered either gradually or quickly dissolving based on their dissolution rate, the relative contribution of the ion and particle components to the toxicity observed during hazard testing will need to be determined.^78^ Also, the potential for the particle and ion to interact to enhance toxicity should be considered.^75^

### Inflammatory potential DNs

The next step for grouping according to the IATA is to assess the potential of the NFs to elicit an inflammatory response compared with the source material. Endpoints for assessing the lung inflammatory potential should be informed by adverse outcome pathways (AOPs) that are relevant for pulmonary disease, to ensure the information gathered is targeted and can be interpreted in terms of disease relevance. Therefore, the Tier 1 and Tier 2 *in vitro* assays could be selected based on the measurable key events outlined in the AOP.^54^ Inflammatory potential can be tested in tiers from simple *in vitro* assays by using cell-lines and acute endpoints (Tier 1), to more complex and physiologically relevant *in vitro* models incorporating multiple cell types and using air–liquid interface (ALI) exposure (Tier 2). If necessary, Tier 3 recommends *in vivo* hazard assessment using an STIS.

Starting at Tier 1, we recommend simple *in vitro* screening assays following well established protocols. The preferred assay measures inflammasome activation in the human monocyte cell-line THP-1 (SOP from REFINE (Vandebriel et al. submitted 2021)). NLRP3 inflammasome activation is an important step in the immune response to NFs,^79^ as it contributes to pulmonary diseases, including asthma, chronic obstructive pulmonary disease, fibrosis, and cancer.^79–81^ Inflammasome activation appears to regulate the balance between tissue repair and inflammation after inhalation of NFs^82^ and is, therefore, key in understanding the inflammation potential of NFs. Several NFs have been shown to activate the NLRP3 inflammasome, including Ag, CeO_2_, carbon nanotubes, polystyrene, TiO_2_, and SiO_2._^83^ Another suitable Tier 1 *in vitro* assay that can be used to assess macrophage activation and inflammatory potential of NFs is based on rat alveolar macrophages (NR8383). According to two recent publications, NR8383 assay outcome showed reasonable predictivity to *in vivo* STIS for more than twenty NFs, including AlOOH, BaSO_4_, different CeO_2_, Fe_2_O_3_, TiO_2_, different nano ZrO_2_, and ZnO, different amorphous SiO_2_ and graphite nanoplatelets, and two nanosized organic pigments.^84,85^

Submerged exposure can greatly alter particle characteristics compared with the airborne state. Therefore, at Tier 2 we recommend using ALI exposure to mimic inhalation exposure more closely.^86^ Several researchers have shown that using ALI exposure improves the predictive value of *in vitro* systems.^87–91^ Another way of enhancing predictivity is to better mimic physiological relevance of the *in vitro* model by using co-cultures or tissue models cultured from primary cells. The downside of these more complex models is that these methods have not been validated or standardized and are undergoing constant optimizations to allow better predictions.^86^ SOPs and publications^90,92^ from the H2020 project PATROLS (https://www.patrols-h2020.eu), provide useful information toward improved standardization of these methods.

As inflammation is a complex process, Tier 3 STISs^93^ might be required to substantiate a read-across argument. If a target NF and a source material show similar potency in a short-term study, this can be used to substantiate a read-across argument for the hazards after repeated exposure from the source to the target NF. We recommend that if *in vivo* studies are considered, inclusion of Tier 3 measurements for all DN (dissolution and reactivity) is combined within one study to avoid additional *in vivo* testing for the other DN. STIS should be performed following the recommendation of OECD Guidance Document 39.^94^ Nose-only is the most preferred exposure mode.^95^ In case that a study according to OECD test guidelines^72,73^ is required later on for regulatory purposes, the STIS data can assist the scientist to appropriately design their regulatory study.

## Demonstration of IATAs

Based on the information gathered on each DN, similarity can be assessed between the target NF and the source material. Depending on the purpose, this similarity assessment can be qualitative or quantitative.

- Qualitative: use the IATAs to gather the evidence required to assess whether NFs are sufficiently similar to be grouped. Qualitative similarity assessment may be based on information from a variety of assays deemed appropriate to answer the IATA, justified by expert opinion. Qualitative similarity assessment based on expert judgement can help r by Design-by-Design, and it is the first step for regulatory read-across. Based on such qualitative similarity, precautionary measures can be taken in the workplace.
- Quantitative: based on the outcome of the qualitative similarity assessment, perform a detailed quantitative similarity assessment employing mathematically derived limits of similarity between group members within each individual assay of a DN to support read-across to fill a data gap.

The IATA as presented here directs the collection of the minimum relevant evidence needed to conduct similarity assessment to confirm the proposed substances/NFs can be grouped, and to subsequently support any read-across arguments relevant to the hypothesis. Later, we focus on qualitative assessment of the similarity between NFs.

### Selection of source materials

To form a preliminary group, a source material first needs to be selected against which the NF under investigation is compared. There are several considerations for selecting a source material (or materials), which depends on the purpose of grouping. For SbD and for adopting precautionary measures, less detailed information on similarity is needed. In this case, the target NF can be compared with a data-rich benchmark material such as the reference materials from the Joint Research Centre (JRC) repository. These benchmark materials can also serve as positive and negative controls to indicate the maximum and minimum responses in an assay. To be considered acceptable for regulatory read-across, a high level of similarity is needed to justify filling a data gap using information from a source material. In this case, the source material should be of similar chemical composition. For example, NFs that differ in morphology or coating can be compared, or the target NF can be compared with its bulk non-nano counterpart.

### Benchmark material for the IATA on very slowly dissolving NFs: CeO_2_

CeO_2_ NFs are widely distributed, as they are used as polishing materials, absorbents, exhaust catalysts, conductors, and electrode materials. CeO_2_ NFs are known to have a very slow dissolution rate. As a case study, we selected two well-characterized reference materials, JRCNM02102a (formerly known as NM-212) and JRCNM02101a (formerly known as NM-211), supplied by the JRC. Both NFs of CeO_2_ are uncoated and produced by precipitation; however, these NFs of CeO_2_ differ in size and morphology. Table 2 shows some key characteristics of JRCNM02102a and JRCNM02101a reported by the JRC.^96^ JRCNM02102a, in particular, has been studied extensively, including long-term inhalation studies; such studies are lacking for JRCNM02101a. JRCNM02102a is known to have a half-life >60 days.^97^ Results from 90-day inhalation studies show that JRCNM02102a can accumulate in the lungs on subchronic exposure, leading to chronic inflammation and fibrosis, therefore as JRCNM02102a is considered a very slowly dissolving NF that can induce long-term effects. H-I-S was selected as the most appropriate pre-defined hypothesis for potentially grouping different NFs of CeO_2._

**Table 2.** Particle Characteristics of Joint Research Center Materials JRCNM02102a and JRCNM02101a

	Primary particle size	Specific surface area	Morphology from TEM image
NM-211	<10 nm up to 20 nm	27.8 ± 1.5 m^2^/g	Spherical with regular morphology
NM-212	<10 nm up to 100 nm	64.9 ± 4.1 m^2^/g	Polyhedral with irregular morphology and non-homogenous size distribution

NM, nanomaterial; TEM, transmission electron microscopy.

The aim of the case study exercise was to assess whether the IATA can be used to support the grouping of JRCNM02102a and JRCNM02101a on the basis of a common fate and hazard potential, despite certain dissimilarities between the NFs as highlighted in Table 2. The potential IATA outcomes for this case study are outlined in Box 1. Following IATA for the hypothesis on very slowly dissolving NFs, data were gathered to address each DN (Tables 3 and 4).

**Table 3.** Data Matrix for JRCNM02102a as a Benchmark Material for Very Slowly Dissolving Nanoforms

Tier	NM212		
	Dissolution	Reactivity	Inflammation potential
1	Flask dialysis <1 μg/L.^96^ Static in PLF <0.001 Wt % (recrystallizing).^65,97^ Dynamic in PLF: <0.28 ng/cm^2^/h.^65^ Half-time >365 days (Wohlleben et al. 2021 in prep)	FRAS: 16.7 sBOD at 1000 m^2^/L.	Inflammasome activation: at 10–30 μg/cm^2^.^100^ Submerged exposure: increased TNF-α in NR8383 at 22.5 μg/mL.^84^
2		ALI exposure: no oxidative stress observed up to 3 μg/cm^2101^; Submerged in co-culture: no oxidative stress up to 10 μg/m^2^.^101^	ALI exposure: no release of cytokines up to 5 μg/cm^2100^; ALI exposure: increased IL-6 and IL-1β at 1–3 μg/cm^2101^; Submerged in co-culture: Increased IL-1β, IL-6, IL-8 and TNF-α at 10 μg/cm^2^.^101^
3	5 and 28 days study: T ½ 40 days at 0.5 mg/m^3^, T ½ > 200 days at >5 mg/m^3^.^97^ Instillation: T ½–140 days at 1 mg/kg bw.^102^ 28 days study: no significant reduction of CeO_2_ content in lung and extrapulmonary organs at 48 and 72 hours after exposure to 20 mg/m^3^.^103^ 90 days study: impaired clearance at 3 mg/m^3^.^104^ 2-year study: T ½ 86, 114, 164 and 200 days at 0.1, 0.3, 1.0 and 3.0 mg/m^3105^	28 days study: oxidative stress (8-OH-dG) not demonstrated at 20 mg/m^3^.^98^ 90 days study: increased expression of oxidative stress-related genes at 3 mg/m^3^,^106^ increased 8-OH-dG at 3 mg/m^3^.^61^	5 days study: increased neutrophils in lavage fluid at 0.5 mg/m^3^,^97^ 28 days study: granulomatous inflammation at 5 and 25 mg/m^397^; increased neutrophils at 2.5 mg/m^3^.^98^ 90 days study: neutrophilic infiltration and granulomatous inflammation at 3 mg/m^3^, progression to fibrosis.^104^
Evaluation	Very slowly dissolving *in vitro*; accumulation and very slow clearance *in vivo.*	No oxidative stress *in vitro* in cells; ambiguous results *in vivo.*	Induction of cytokines *in vitro* and inflammation *in vivo.*

8-OH-dG, 8-hydroxy-2-deoxyguanosine; ALI, air–liquid interface; CeO_2_, cerium dioxide; FRAS, ferric reduction ability of serum; IL, interleukin; PLF, phagolysosomal fluid; TNF-α, tumor necrosis factor α.

**Table 4.** Data Matrix for JRCNM02101a to Test the Integrated Approaches to Testing and Assessment for Very Slowly Dissolving Nanoforms

Tier	NM211		
	Dissolution	Reactivity	Inflammation potential
1	Flask dialysis <1 μg/L. Static in PLF <0.001 Wt % (recrystallizing).^97^ Dynamic in PLF: <0.73 ng/cm^2^/hour.^65^ Half-time >365 days (Wohlleben et al. 2021 in prep)	FRAS:13 sBOD at 1000 m^2^/L.	Submerged: increased TNF-α in NR 8383 at 22.5 μg/mL.^84^
2			
3	5 and 28 days study: high lung burden 3 weeks after exposure to 25 mg/m^3^.^65,107^ 28 days study: no significant reduction of CeO2 content in lung and extrapulmonary organs 48 and 72 hours after exposure to 10 mg/m^3^.^103^	28 day study: oxidative stress (8-OH-dG) not demonstrated at 10 mg/m^3^.^98^	5 day study: increased neutrophils in lavage fluid at 0.5 mg/m^3^.^97^ 28 day study: increased neutrophils at 1.2 mg/m^3^.^98^
Evaluation	Very slowly dissolving *in vitro*; accumulation and very slow clearance *in vivo.*	Little information available. No oxidative stress observed *in vivo.*	Induction of cytokines *in vitro* and inflammation *in vivo.*

Following the DN in the IATA, we can perform a qualitative similarity assessment to compare the two CeO_2_ NFs (Table 5). From the available data, it is clear that both NFs are very slowly dissolving and have the potential to accumulate in lung tissues after inhalation exposure. This might lead to long-term effects on repeated exposure. A limited number of studies were identified reporting on the reactivity of JRCNM02102a and JRCNM02101a; however from this data set, neither of the NFs appears to intrinsically produce high levels of ROS or induce significant oxidative stress *in vitro* or in short-term *in vivo* studies. JRCNM02102a exposure, however, resulted in increased expression of oxidative stress-related genes and increased 8-OH-dG after 90-day inhalation.^61^ Both JRCNM02102a and JRCNM02101a were shown to induce pro-inflammatory responses in simple *in vitro* assays, which was reflected in the development of acute and persistent inflammation *in vivo* after short-term inhalation exposure.^97,98^ Therefore, the hypothesis that both JRCNM02102a and JRCNM02101a can be grouped as slowly dissolving NF with the potential to cause long-term toxicity in the lung can be accepted.

**Table 5.** Comparison of JRCNM02102a and JRCNM02101a Based on the Integrated Approaches to Testing and Assessment Following the Hypothesis for Very Slowly Dissolving Nanoforms

IATA DN	JRCNM02102a	JRCNM02101a
Dissolution	Very slowly dissolving *in vitro*; accumulation and very slow clearance *in vivo.*	Very slowly dissolving *in vitro*; accumulation and very slow clearance *in vivo.*
Reactivity	Little information available. No oxidative stress observed *in vivo* after 28 days of exposure, whereas oxidative stress was observed *in vivo* after 90 days of exposure.	Little information available. No oxidative stress observed *in vivo* after 28 days of exposure.
Inflammation	Induction of cytokines *in vitro*; inflammation *in vivo* (5 and 28 days of exposure).	Induction of cytokines *in vitro*; inflammation *in vivo* (5 and 28 days of exposure).
IATA outcome	Accept hypothesis: after chronic inhalation exposure, accumulation of NFs in the lungs can lead to long-term toxicity. Form group, for SbD and for adopting precautionary measures: Assume NM211 can cause similar toxicity compared to NM212 upon long-term exposure.	

IATA, Integrated Approaches to Testing and Assessment.

For the purpose of SbD or for adopting precautionary measures, the acceptance of the grouping hypothesis supports the prediction that JRCNM02101a can induce impaired clearance and granulomatous inflammation that can progress to fibrosis as reported for JRCNM02102a after 90 days of inhalation exposure.

### Benchmark material for the IATA on quickly dissolving NFs: ZnO

Zinc Oxide NFs (ZnO) was chosen as a case study material to exemplify the substantiation of the pre-defined hypothesis, H-I-Q. We collected data relevant to each DN for a single specific ZnO NF, JRCNM01100a (formerly known as NM-110) (Table 6).

**Table 6.** Data Matrix for ZnO JRCNM01100a as a Reference Material for Quickly Dissolving Nanoforms

Tier	Dissolution	Reactivity	Inflammation potential
1	Static system: <0.05% dissolution in LLF, >90% dissolution in PLF.^108^ Static system: 67% dissolution in PLF.^65^ Dynamic system: K_diss_: 204 ng/cm^2^/hour in PLF complete dissolution confirmed after 7 days by TEM.^65^	FRAS assay: intermediate reactivity.^108^	Submerged: increased production of TNF-α and IL-8 in THP-1.^109^ Submerged: increased IL-8 in human hepatoblastoma C3A cells:^110^ Submerged: increased IL-8 and MCP-1 in dHL-60 neutrophil cell.^111^ Submerged: increased levels of TNF-α production in HMDM.^112^
2	Cellular: 51% dissolution after 24 hours in NR8383 macrophages.^65^ Cellular: complete dissolution after 24 hours in THP-1.^109^	Cellular, submerged: dose-dependent decrease in reduced GSH and total glutathione antioxidant in human hepatoblastoma C3A cells.^113^ p47^phox^ NADPH oxidase-mediated ROS formation in RAW 264.7.^114^] DCFH_2_-DA cellular: ROS release in 16HBE cells.^112^ Cellular, submerged: Upregulation of HSP genes at 4 hours in THP-1.^115^	Submerged: modifications of genes involved in inflammation, apoptosis, and mitochondrial dysregulation at 4 hours in THP-1.^115^ Submerged: severe tissue destruction at 10–1000 μg/mL at 24 hours in rat precision-cut lung slices.^116^ Molecular responses of A549 cells measured by multiple “omics” platforms at 24 hours: metallothionein induction, depletion of antioxidants, repressed DNA repair, and induction of apoptosis. Responses to NM110 similar to Zn^2+^ ions, suggesting that the mode of action is mediated by dissolved metal ions rather than by the physical NF.^117^
3	IT: No ZnO NM agglomerates observed inside the BAL macrophages after 24 hours.^118^		IT in mouse: increased total number, IL-6, LDH, and protein in lavage fluid at 64 and 128 μg/mouse.^118^ IT in mouse: increased acute-phase response at 11, 33, and 100 mg/kg bw.^119^.
Evaluation	Quick dissolution in the low pH acellular assays. Evidence of quick dissolution within cells after uptake. No accumulation *in vivo*.	Reactive in acellular assays and cellular assays.	Induced pro-inflammatory signaling *in vitro*. Acute resolving inflammation *in vivo*. Toxicity is driven by intracellular release of toxic ions rather than particle-driven.

BAL, bronchoalveolar lavage; DCFH_2_-DA, dichlorodihydrofluorescin diacetate assay; GSH, glutathione; HMDM, human monocyte-derived macrophages; HSP, heat-shock proteins; IT, intratracheal instillation; LDH, lactate dehydrogenase; LLF, lung lining fluid; MCP-1, monocyte chemoattractant protein-1; NADPH, nicotinamide adenine dinucleotide phosphate; ROS, reactive oxygen species; THP-1, human monocytic cell line.

Evidence of dissolution rate is sufficient to identify JRCNM01100a as quickly dissolving, and the reactivity and inflammatory data suggest that toxicity is driven by the intracellular release of toxic ions rather than the NF itself. Data from studies conducted on other forms of ZnO NFs further support the conclusion that ZnO NFs can be considered as quickly dissolving NFs with minimal potential for accumulation. Accordingly, hazard results from the intracellular dissolution of ZnO NFs to toxic ions have been demonstrated by both *in vitro* and *in vivo* models.^56,58^ Grouping via H-I-Q will, therefore, allow the similarity assessment between NFs to be framed by the likely relevant mechanism of action driving the potential hazard. Available *in vivo* data for JRCNM01100a consist of two IT studies. The IT studies have major shortcomings, as, for example, it is very difficult with IT to get a material spread evenly among the lung lobes and all material could end up in a single lobe and by-pass the upper respiratory tract. In addition, usually unrealistically high exposure doses are being used for IT, leading to a bolus effect regardless of the toxicity of NFs.^95^ Therefore, IT is not considered a physiological route of exposure. However, NFs with high toxicity have been shown to induce persistent inflammation, whereas NFs with low toxicity induced only transient inflammation after IT. IT could be useful for screening for hazard of NFs.^95^

## Discussion

Here, we present a range of inhalation grouping hypotheses, which are evidence based, employing knowledge from a wide range of published data. In addition, we present a novel tailored IATA supported by a tiered testing strategy to provide the evidence needed to support, reject, or refine these grouping hypotheses. Each hypothesis takes into consideration the PC characteristics of the NFs (what they are), the route of exposure and toxicokinetics (where they go), and their hazard (what they do). For the PC characteristics, dissolution rate was found to be an efficient mechanism by which to group NFs, as this determines their biopersistence and their fate and behavior. Coupling the biopersistence with assessment of the hazard in terms of surface reactivity and pro-inflammatory potential allows further refinement of the group.

Thresholds were provided for the dissolution rate based on biologically relevant timeframes for cell interaction and cellular clearance from the lungs. Clearly, particles that dissolve instantaneously (t_1/2_ < 10 minutes) in LLF will not persist for sufficient time to induce particle-mediated effects. For this reason, hypothesis H-I-I supports the argument to read-across from the ionic or molecular form of the same substance to a NF. In contrast, particles that are very slow to dissolve (t_1/2_ > 60 days) may induce particle-mediated toxicity and bioaccumulate (H-I-S),^24–26^ with the potential to cause longer-term hazards. For the particles that have intermediary dissolution, the toxicity could be driven by particles and/or dissolution products. The rate of release of dissolution products will influence the rate at which these products are released in the cell and so their toxic potential, as well as the duration of particle persistence in the cell and so any biological effects imparted by the residual particles. We, therefore, set two thresholds, one for gradual dissolution with a half-life of >48 hours in lysosomal fluid (H-I-G) for which accumulation cannot be discounted, and one for quick dissolution with a half-life of <48 hours in lysosomal fluid (H-I-Q). However, these values are not strictly fixed. Values close to the thresholds can be supported by use of a similarity assessment.

The remaining wording of each hypotheses is less well prescribed, to allow flexibility. Instead, the evidence generated by use of the IATA provides the more precise details required to define a group, and it can be tailored to support read-across for a specific hazard endpoint, for example, repeated dose toxicity after inhalation exposure. For example, the hypothesis for particles that dissolve quickly could be used to group particles with very low reactivity, or alternatively to group particles with relatively high reactivity. For regulatory purposes, the need to provide thresholds for such descriptors is prevented by incorporation of robust and quantitative methods of assessing similarity (Jeliazkova et al. 2021 in preparation).

The IATA includes DN on reactivity and inflammation potential for assessing similarity between the target NF and a source material. Surface reactivity and inflammation potential are included, as both are considered key toxicity parameters for NFs after inhalation exposure.^5,8,9,24,49–52^ They are both associated with pathological outcomes: Oxidative stress is associated with genotoxicity and inflammation,^36–40^ and inflammation is linked with pulmonary fibrosis and cancer.^25,54^ A key toxicity parameter that is currently not included in the IATA is genotoxic potential. The main reason for not including this here is that current *in vitro* assays for testing genotoxic potential need modifications before they can be used to test NFs.^99^ Experts from the Genetic Toxicology Technical Committee (GTTC) critically reviewed published data on genotoxicity assessment of NFs and found large variation in tests and systems used for *in vitro* assays. They concluded that these results cannot be interpreted and first modifications of the current *in vitro* assays are needed.^99^ In addition, the experts of GTTC conclude that it appears that genotoxicity by NFs is mainly induced via a secondary effect (such as via oxidative stress and/or chronic inflammation) and not via direct DNA interaction. Based on the recommendations by GTTC, a testing strategy for assessing the genotoxic potential of an NF is being developed. The IATAs presented here to support grouping and read-across of NMs after inhalation exposure can then be updated accordingly with a DN on genotoxicity.

Also, systemic toxicity in secondary organs and local toxicity within the upper respiratory tract are not specified in the IATA. As stated earlier, respirable particles are the focus of our IATA. However, the particle sizes of NFs usually cover a range and they will be deposited within the entire respiratory tract depending on the aerodynamic size distribution. At the deposited site, for example, nasal cavity or larynx, NFs may cause local toxicity. For instantaneously, quickly, and gradually dissolving NFs, the local toxicity of the released ions can be assessed by read-across to the ionic or molecular form. Potential particle-triggered toxicity at the upper respiratory tract may be assessed by particle surface reactivity and inflammation potency. Finally, local toxicity to the upper respiratory tract can be assessed in Tier 3 STIS. Thus, this point is covered by the IATA.

Systemic toxicity can occur in the case of translocation of the NFs or their ions to the blood. For instantaneously, quickly, and gradually dissolving NFs, the released ions might translocate to the blood. For these NFs, read-across to the ionic or molecular form can be performed for assessing systemic toxicity. For very slowly dissolving NFs, translocation of the particles depends strongly on their physical–chemical properties and the region of deposition. The NFs deposited in the upper respiratory tract will be cleared via mucociliary transport and are subsequently swallowed and cleared via the gastrointestinal tract. The NFs that deposit in the alveoli might translocate to the blood. The translocation and systemic toxicity in secondary organs can only be assessed in *in vivo* inhalation studies. Because the existing data of repeated dose inhalation studies with very slowly dissolving NFs did not give indication for any systemic toxicity in secondary organs, and there were no established Tier 1 and Tier 2 tests available, we decided not to include systemic toxicity in our IATA.

The IATAs have been proposed by the OECD for streamlining of information gathering and testing for hazard assessment of chemicals. In the context of GRACIOUS, we have used them to streamline the evidence identification and generation to test specific grouping hypotheses. We assessed the suitability of the hypotheses and the IATA through application of case studies. The case studies included CeO_2_ JRCNM02102a, CeO_2_ JRCNM02101a, and ZnO JRCNM01100a, for which much data are available and that we propose as benchmark materials. Such benchmark materials will be useful for comparison to the NF of concern or for identifying the range of biological relevance (maximal or minimal biological response) for a particular descriptor. The data identified via the IATA were gathered into a matrix, providing insight into data gaps for these benchmark materials. The IATA starts with a DN addressing dissolution to identify the most relevant of the hypotheses. The CeO_2_ NFs have a very slow dissolution rate, relevant to H-I-S, whereas ZnO exhibits quick dissolution, relevant to H-I-Q. Regarding dissolution, sufficient data are available for Tier 1 to assess the relevant thresholds, but little data are available on Tier 2 assays (dissolution in cells). Such Tier 2 methods are quite laborious and do not provide much added value compared with the dynamic acellular dissolution assay. We, therefore, suggest that in many instances the Tier 1 assays are sufficient for assessment of dissolution in relation to grouping. A Tier 2 assessment of dissolution may be more relevant to the hypotheses where gradual or quick dissolution intracellularly is relevant (H-I-Q and H-I-G).

For the other DNs, sufficient data were observed for Tier 1 assays and for Tier 3 *in vivo* studies, whereas there were limited data available for Tier 2 assays. The proposed Tier 2 assays are generally of higher complexity than Tier 1 assays, plus they are relatively innovative and therefore lack standardization. During application of the IATA during grouping, Tier 3 *in vivo* data might be lacking for some NFs. Tier 2 data could, therefore, be needed to provide the data required for a similarity assessment between NFs within the group. Innovative Tier 2 assays, such as co-cultures, primary cells, and/or exposure at the ALI, may be more predictive due to a higher physiological relevance, or by allowing identification of the mechanism triggering toxicity. For example, some ALI models show a better correlation to *in vivo* data than submerged models.^87–91^ The disadvantage of more complex models, at this time, is that optimizations are ongoing and therefore standardization is currently lacking. For grouping purposes, it would be ideal to have an assay that is simple and predictive at the same time. Tier 2 assays will require optimization to deliver this need. In future, as Tier 2 assays are validated and evidence builds to demonstrate that such assays are suitably and reliably predictive of hazards, the waiving of Tier 3 *in vivo* testing may be justified, reducing the reliance on animal testing for NM hazards.

As described earlier, once the data are collected into a matrix a qualitative or quantitative similarity assessment can be conducted. Qualitative approaches can be used to inform the SbD of NFs, or for adopting precautionary measures. For regulatory read-across, a quantitative similarity assessment between group members is needed. For the purpose of read-across to fill a data gap for regulatory hazard assessment, such as extrapolation of the 90-day inhalation study point of departure from JRCNM02102a to JRCNM02101a, a read-across argument will need to be built. This will require a quantitative similarity assessment to compare the potencies of the target (JRCNM02101a) and the source NF (JRCNM02102a), based on the available data gathered using the IATAs. Several methods of quantitative similarity assessment have been generated and will form the basis of a White Paper and a further 12 publications to be published in NanoImpact (to be submitted by June 2021). A full description of these methods is, therefore, beyond the scope of this article.

The grouping approach and IATAs presented here will be integrated in the overall GRACIOUS framework.^10^ The GRACIOUS framework will guide the user through the different steps to hypothesis selection and subsequent IATA testing to allow grouping.^5–9^ The GRACIOUS framework will be available as a guidance document and also as a software “blueprint” tool (to be published Sept 2021). Linked to NM databases (e.g., eNanoMapper), the open-access blueprint will facilitate the rapid identification of potential group members or potential source materials and provide a user-friendly interface to facilitate the use of the IATA to support grouping and subsequent read-across.

Grouping approaches are necessary to perform risk assessment based on limited data. The GRACIOUS approach presented here provides an intuitive way to group NFs based on hypotheses and using an IATA that guides the user to an outcome. We believe that this approach is a step forward to streamline hazard assessment of NFs and hope it will be expanded in the future to allow growth of safe nanotechnology.

## Supplementary Material

Supplemental data
