# Supplementary material for: An Integrated Approach to Testing and Assessment to Support Grouping and Read-Across of Nanomaterials After Inhalation Exposure
Source: Appl In Vitro Toxicol. 2021 Sep 17;7(3):112–28. doi: 10.1089/aivt.2021.0009 (PMC8567336; doi:10.1089/aivt.2021.0009)
Supplement: Supplemental data [file Supp_Table1.docx]

Table S1. Definition of a nanomaterial and a nanoform.

| **Term** | **Explanation** |
| --- | --- |
| Nanomaterial  [1] | A natural, incidental or manufactured material containing particles, in an unbound state or as an aggregate or as an agglomerate and where, for 50 % or more of the particles in the number size distribution, one or more external dimensions is in the size range 1 nm - 100 nm. […]. By derogation from the above, fullerenes, graphene flakes and single wall carbon nanotubes with one or more external dimensions below 1 nm should be considered as nanomaterials.  For this purpose, “particle” means a minute piece of matter with defined physical boundaries; “agglomerate” means a collection of weakly bound particles or aggregates where the resulting external surface area is similar to the sum of the surface areas of the individual components and “aggregate” means a particle comprising of strongly bound or fused particles. |
| Nanoform  [2] | On the basis of the Commission Recommendation of 18 October 2011 on the definition of nanomaterial, a nanoform is a form of a natural or manufactured substance containing particles, in an unbound state or as an aggregate or as an agglomerate and where, for 50 % or more of the particles in the number size distribution, one or more external dimensions is in the size range 1 nm-100 nm, including also by derogation fullerenes, graphene flakes and single wall carbon nanotubes with one or more external dimensions below 1 nm. […]  A nanoform shall be characterised in accordance with REACH Annex VI, section 2.4. A substance may have one or more NFs, based e.g. on differences in their number based particle size distribution, shape, aspect ratio, crystallinity, assembly structure, specific surface area and surface functionalisation or treatment (REACH Annex VI, points 2.4.2. – 2.4.5).[2] |

1. European_Commission, *EU Commission recommendation on the definition of nanomaterial. OJ L 2011;275:38*. 2011.

2. Commission, E., *Commission Regulation (EU) 2018/1881 of 3 December 2018 amending Regulation (EC) No 1907/2006 of the European Parliament and of the Council on the Registration, Evaluation, Authorisation and Restriction of Chemicals (REACH) as regards Annexes I, III,VI, VII, VIII, IX, X, XI, and XII to address nanoforms of substances (Text with EEA relevance.)*, in *C/2018/7942*, E. Commission, Editor. 2018.
